# Misdiagnosis of HIV treatment failure based on clinical and immunological criteria in Eastern and Central Kenya

**DOI:** 10.1186/s12879-017-2487-5

**Published:** 2017-06-02

**Authors:** Sunguti Luke Joram, Gathii Paul, Kitheka Moses, Bii Stanley, Malonza Isaac, Gohole Allan, Marwa Tom, Karimi Lilian, Mudany Mildred

**Affiliations:** 1 APHIAPLUSKAMILI, P.O. Box 2373, Embu, Kenya; 2 Jhpiego, an affiliate of Johns Hopkins University, Nairobi, Kenya; 3 USAID, Nairobi, Kenya; 4 Ministry of Health, Meru County, Kenya

**Keywords:** HIV, Viral load testing, Routine monitoring, Kenya

## Abstract

**Background:**

Routine laboratory monitoring is part of the basic care package offered to people living with the Human Immunodeficiency Virus (PLHIV). This paper aims to identify the proportion of PLHIVs with clinical and immunological failure who are virologically suppressed and risk being misclassified as treatment failures.

**Methods:**

A retrospective analysis of patient viral load data collected between January 2013 and June 2014 was conducted. Of the patients classified as experiencing either clinical or immunological failure, we evaluated the proportion of true (virological) failure, and estimated the sensitivity and specificity of the immunological and clinical criteria in diagnosing true treatment failure.

**Results:**

Of the 27,418 PLHIVs aged 2–80 years on ART in the study period, 6.8% (*n* = 1859) were suspected of treatment failure and their viral loads analysed. 40% (*n* = 737) demonstrated viral suppression (VL < 1000 copies/ml). The median viral load (VL) was 3317 copies/ml (IQR 0–47,547). Among the 799 (2.9%) PLHIVs on ART classified as having clinical failure, 41.1% (*n* = 328) of them had confirmed viral suppression. Of the 463 (1.7%) classified as having immunological failure, 36.9% (*n* = 171) had confirmed viral suppression. The sensitivity of the clinical criteria in diagnosing true failure was 61% (CI 58%–65%) while that of the immunological criteria 38% (CI 35%–42%). The specificity of the clinical criteria was 34% (CI 30%–39%) while that of the immunological criteria 66% (61%–70%). Age below 20 years was associated with a high viral load (*p* < .001). Sex and ART regimen were not associated with the viral load.

**Conclusion:**

Clinical and immunological criteria alone are not sufficient to identify true treatment failure. There is need for accurate treatment failure diagnosis through viral load testing to avoid incorrect early or delayed switching of patients to second-line regimens. This study recommends increased viral load testing in line with the Kenya’s ART guidelines.

## Background

In 2015, an estimated 1.5 million Kenyans were living with HIV, with 897,000 of them on antiretroviral therapy (ART) [1]. The number of HIV-infected Kenyans accessing ART is expected to rise due to increased early identification of HIV, roll out of the new ART guidelines [2], and increased adherence and retention in line with the UNAIDS 90–90-90 strategy, which has been adopted wholeheartedly by the Kenyan government. Whilst a majority of patients on ART are expected to achieve viral suppression within one year of treatment initiation [3, 4], 5–21% will experience treatment failure within 5 years of initiation [5–7]. Timely identification of true treatment failure in patients on ART is vitally important as it informs optimization of the use of second-line regimens and protects against unnecessary switching of ART. The Kenya Ministry of Health (MOH) recommends the use of a combination of Tenofovir (TDF)/ Zidovudine (AZT)/Abacavir (ABC), Lamivudine (3TC) and Nevirapine (NVP)/ Efavirenz (EFV) as the first-line ART regimen, with Atazanavir (ATV), Lopinavir and Ritonavir (LPV/r) currently reserved for second-line use.

Many organizations work jointly with the MOH in addressing the HIV epidemic in Kenya. The USAID-funded APHIA*PLUS* KAMILI is one such project, supporting HIV service delivery in Eastern and Central Kenya, a catchment area of 11 counties and over nine million inhabitants. The project supports 46,264 PLHIVs, of whom 34,648 are on ART. Integrated service delivery focuses on HIV testing services (HTS), care and treatment, prevention of mother-to-child transmission (PMTCT), orphaned and vulnerable children (OVC) services, and reproductive health activities. A critical project component is laboratory networking to ensure timely collection and transport of viral load samples and a rapid turn-around time for results.

In Kenya, routine laboratory monitoring is part of the basic care package offered to people living with HIV (PLHIV), and includes tests to monitor the efficacy of ART on viral suppression (CD4 and viral load). Clinical failure is defined as occurrence of a new or recurrent WHO stage 3 or 4 disease after at least six months on ART (Table 1), while immunological failure refers to a CD4 count decrease by >30% from peak or failure of CD4 count to rise to >100 cells/mm^3^ after 12 months on ART [8]. Virological failure occurs when the repeat viral load remains persistently above 1000 copies/ml after three months of adherence counselling. The Kenyan Ministry of Health currently recommends the use of virological monitoring to identify treatment failure for patients on ART, with CD4 testing reserved for baseline investigation. The Ministry of Health has fully adopted routine viral load monitoring and disregarded the use of viral load for confirmatory testing in all health facilities offering HIV care and treatment in Kenya.

**Table 1:** WHO staging

WHO clinical stage 1
1. Asymptomatic
2. Persistent generalized lymphadenopathy
WHO clinical stage 2
1. Moderate unexplained weight loss (<10% of presumed or measured body weight).
2. Recurrent respiratory tract infections (RTIs, sinusitis, bronchitis, otitis media, pharyngitis).
3. Herpes zoster
4. Angular cheilitis
5. Recurrent oral ulcerations
6. Papular pruritic eruptions
7. Seborrhoeic dermatitis
8. Fungal nail infections of fingers
WHO clinical stage 3
1. Conditions where a presumptive diagnosis can be made on the basis of clinical signs or simple investigations.
2. Severe weight loss (>10% of presumed or measured body weight).
3. Unexplained chronic diarrhea for longer than one month.
4. Unexplained persistent fever (intermittent or constant for longer than one month).
5. Oral candidiasis.
6. Oral hairy leukoplakia.
7. Pulmonary tuberculosis
8. Severe presumed bacterial infections (e.g. pneumonia, empyema, pyomyositis, bone or Joint infection, meningitis, bacteraemia).
9. Acute necrotizing ulcerative stomatitis, gingivitis or periodontitis.
10. Conditions where confirmatory diagnostic testing is necessary
• Unexplained anaemia (<8 g/dl), and or neutropenia (<500/mm3) and or
• Thrombocytopenia (<50,000/ mm3) for more than one month.
WHO clinical stage 4 Conditions where a presumptive diagnosis can be made on the basis of clinical signs or simple investigations
1. HIV wasting syndrome
2. Pneumocystis pneumonia
3. Recurrent severe or radiological bacterial pneumonia
4. Chronic herpes simplex infection (orolabial, genital or anorectal of more than one month’s duration)
5. Oesophageal candidiasis
6. Extrapulmonary TB
7. Kaposi’s sarcoma
8. Central nervous system toxoplasmosis
9. HIV encephalopathy
Conditions where confirmatory diagnostic testing is necessary:
1. Extrapulmonary cryptococcosis including meningitis
2. Disseminated non-tuberculous mycobacteria infection
3. Progressive multifocal leukoencephalopathy
4. Candida of trachea, bronchi or lungs
5. Cryptosporidiosis
6. Isosporiasis
7. Visceral herpes simplex infection
8. Cytomegalovirus infection (retinitis or of an organ other than liver, spleen or lymph nodes)
9. Any disseminated mycosis (e.g. histoplasmosis, coccidiomycosis, penicilliosis)
10. Recurrent non-typhoidal salmonella septicaemia
11. Lymphoma (cerebral or B cell non-Hodgkin)
12. Invasive cervical carcinoma
13. Visceral leishmaniasis

In this paper, we analyse viral load data collected from patients with suspected treatment failure based on clinical and immunological criteria between January 2013 and June 2014 from 11 APHIAPLUS KAMILI counties. We aim to demonstrate that a proportion of patients with clinical and immunological failure are virologically suppressed and yet may be misclassified as treatment failure.

## Methods

### Study design

This was a retrospective cross sectional analysis of secondary de-identified data collected for programmatic purposes as part of routine patient care.

### Setting

The study was conducted in eight counties covered by the APHIA*PLUS* KAMILI project, which supports HIV care and treatment in 142 MOH and faith-based organizations health facilities.

### Participants

De-identified electronic medical records data was collected from PLHIVs who had been on ART for more than 6 months. This data was retrieved from the national electronic archive on viral load testing. Patients on second-line ART regimen and those with incomplete socio-demographic and viral load data (597) were excluded from data analysis.

From January 2013 to June 2014, samples from 1859 patients with suspected treatment failure were collected and submitted to the laboratory for viral load testing.

### Laboratory procedures

During program implementation, all PLHIVs suspected of treatment failure had blood drawn for viral load testing as part of their routine laboratory monitoring while on ART. Assays used for the viral load were *Abbott®* RT-PCR (Real-time polymerase chain reaction) (Rungis, France) for HIV-RNA and *Roche Amplicor* RT-PCR (Maylan, France). Blood samples taken included dried blood spots (DBS) and plasma, and were sent to the Kenya Medical Research Institute (KEMRI) and National HIV Reference Laboratory (NHRL) for processing.

### Variables

The variables of interest included age, sex, duration on ART, ART regimen and justification for the viral load test. The primary outcome was the patient’s viral suppression. Virological failure was defined as VL > 1000 copies/ml, immunological failure as a CD4 fall by >30% from peak or failure of CD4 count to rise to >100 cells/mm^3^ after 12 months of ART, and viral suppression as VL < 1000 copies/ml.

### Data analysis

Statistical analysis was conducted using the software SPSS v20 for Windows. Data was checked for consistency and extreme outliers as part of initial data cleaning. Cross-tabulations, histograms and box plots were used to examine the data. Mean, median and standard deviation were used to describe continuous data while frequencies were used for categorical data. Association between variables was assessed through bivariate and multivariate methods. A *p*-value <0.05 was considered statistically significant.

## Results

Viral load data from 1859 patients in eight of the eleven counties supported by the project were analysed (Table 2). Three counties were excluded because they did not have care and treatment sites supported by the project, hence no data on viral load testing. The patient median age was 38 years (IQR 30–47 years), and majority were female (62%). The median viral load was 3317 copies/ml (0–47,547). The most common ART regimens used at the time of study were AZT/3TC/NVP (34%), TDF/3TC/NVP (26%), and TDF/3TC/EFV (23%). The median duration on ART was 4 years (IQR 2–6 years).

**Table 2:** Baseline characteristics

Variable	Total clients	
	*N* = 1859	%
Sex		
Male	532	28.6
Female	872	46.9
Missing	455	24.5
Age group		
< = 20 (ref)	241	13.0
21–40	582	31.3
41–60	556	29.9
61+	65	3.5
Missing	415	22.3
Type of Sample		
DBS	1653	88.9
Frozen Plasma	188	10.1
Missing	18	1.0
Testing lab		
KEMRI NAIROBI	1676	90.2
AMPATH AND NHRL^a^	183	9.8
County^b^ of residence		
Embu	279	15.0
Meru	792	42.6
Kitui	26	1.4
Kirinyaga	53	2.9
Nyeri	138	7.4
Nyandarua	70	3.8
Kiambu	278	15.0
Murang’a	223	12.0
ARV regimen		
TDF/3TC/EFV	254	13.7
TDF/3TC/NVP	286	15.4
AZT/3TC/NVP	375	20.2
Other	176	9.5
Missing	768	41.3
Duration on ARVs		
< 3 years	552	29.7
4–6 years	482	25.9
7–9 years	181	9.7
Missing	644	34.6

^a^AMPATH 4, NHRL 179

^b^Data missing from Machakos, Makueni and Kitui counties

The most common indication by clinicians for requesting viral load testing was clinical failure (59%), followed by immunological failure (34%), and others (7%).

The proportion of patients with viral suppression was 39.5%, with 60.5% experiencing virological failure. There was no significant difference between the proportion of male patients who were virally suppressed (43.1%) and that of female patients (43.6%). Viral suppression was highest among patients aged above 61 years (55.4%) and lowest in those aged below 20 (23.9%) years.

**Fig. 1:**
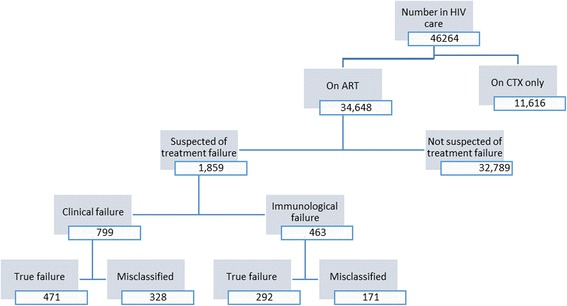
Participant selection

Among the 799 patients who were reported as having clinical failure, 41.1% (*n* = 328) had confirmed viral suppression. Of the 463 patients reported as having immunological failure, 36.9% (*n* = 171) had confirmed viral suppression (Fig. 1). The combined number for both clinical and immunological failure was 1262. Of these, 499 (39.5%) had confirmed viral suppression and had been misclassified.

These results indicate that 41.1% of patients in the clinical failure group were misclassified as having treatment failure, compared to 36.9% patients in the immunological failure group. The difference in proportion of misclassified patients between the two groups was, however, not statistically significant (OR 0.84, CI 0.66–1.06). The sensitivity of clinical criteria in diagnosing true failure was 61% (CI 58%–65%) while immunological criteria had a low sensitivity of 38% (CI 35%–42%). The specificity of the clinical criteria was 34% (CI 30%–39%) while that of the immunological criteria was 66% (61%–70%) (Table 3).

**Table 3:** Sensitivity and specificity

	Sensitivity		Specificity		PPV		NPV	
	%	CI	%	CI	%	CI	%	CI
Clinical Failure	61	58–65	34	30–39	59	55–62	37	33–42
Immunological Failure	38	35–42	66	61–70	63	58–67	41	38–45

At both bivariate and multivariate levels, increase in age of the patient was associated with reduced odds of virological failure (Table 4). Associations between virological failure and sex, ART regimen and duration on ART was not found to be statistically significant.

**Table 4:** Bivariate and multivariate analysis

Factors	COR	*P*-value	95% CI for COR		AOR	*P*-value	95% CI for AOR	
			Lower	Upper			Lower	Upper
Age group								
< = 20 (ref)								
21–40	0.48	<0.001	0.35	0.67	0.48	0.011	0.27	0.84
41–60	0.32	<0.001	0.23	0.45	0.36	0.001	0.20	0.64
61+	0.29	<0.001	0.16	0.50	0.25	0.002	0.10	0.59
Sex								
Male...Ref								
Female	0.99	0.89	0.79	1.22		0.16	0.53	1.11
Sample								
DBS...Ref								
Frozen Plasma	0.82	0.21	0.61	1.11	omitted from the final model			
Testing lab								
KEMRI NAIROBI…Ref					omitted from the final model			
AMPATH AND NHRL	0.78	0.11	0.57	1.06				
ARV regimen								
DF/3TC/EFV...Ref								
TDF/3TC/NVP	1.19	0.33	0.84	1.67	1.49	0.11	0.91	2.42
AZT/3TC/NVP	0.76	0.09	0.55	1.05	0.76	0.23	0.49	1.19
Other	0.85	0.43	0.58	1.26	0.82	0.47	0.49	1.39
Duration on ARVs								
< 3 years…Ref								
4–6 years	1.24	0.09	0.97	1.59	1.31	0.13	0.93	1.87
7–9 years	1.01	0.52	0.72	1.42	0.86	0.53	0.54	1.37
Justification for viral load								
Clinical……Ref								
Immunological	1.19	0.15	0.94	1.51	1.37	0.08	0.96	1.97
Other	0.86	0.48	0.56	1.32	1.06	0.83	0.60	1.88

## Discussion

Viral load monitoring is routinely carried out in PLHIVs in most countries. In low income countries, viral load tests have been previously restricted to drug resistance studies and diagnosis of HIV-exposed infants, possibly due to inadequate resources. In Kenya, viral load testing is now recommended for monitoring of patients on ART, replacing CD4 testing, which has since been reserved for baseline screening to identify patients eligible for cryptococcal meningitis prophylaxis (CD4 < 100).

Our study presents data on discrepancies in classification of treatment failure based on clinical, immunological and virological criteria. Consequently, we demonstrate that the use of clinical and immunological criteria alone may result in misclassification of patients with viral suppression as treatment failure leading to unnecessary, untimely, and incorrect switching to second line regimens.

Our study adds to the literature from studies done elsewhere which also evaluated misclassification of treatment failure in patients on ART. In a prospective cohort study in Uganda, immunological criteria failed to identify all the genuine treatment failures and had a low sensitivity [9]. Studies in Kenya and Uganda by Moor et al. (2008), Kantor et al. (2009) and Mermin et al. (2011) were able to illustrate that virological monitoring is the most accurate way of identifying treatment failure as compared to clinical and/or immunological criteria [10-12]. These studies are important as the use of clinical and immunological criteria alone in treatment failure identification has been associated with increased resistance to HIV [13, 14].

In the course of treatment of PLHIVs, virological failure occurs early, followed by immunological and clinical failure. Therefore, there is need for timely and accurate treatment failure diagnosis based on viral load testing in order to avoid early or delayed switching of patients to second-line ART regimen. Switching early to second-line regimen increases the cost burden of HIV treatment and minimizes options for subsequent regimens, should they be required [11]. On the other hand, delayed switching to a second-line regimen has been associated with increased drug resistance, morbidity and mortality [15].

In this study, the age of the patient was associated with virological failure. Majority of patients who had virological failure fell within the age range of 20–40 years. This data is similar to that of a study conducted in Kenya by Hassan (2014) where young age was a significant predictor for virological failure and drug resistance [16]. Young HIV-infected patients constitute a special cohort experiencing challenges such as stigma, peer pressure, adherence and discrimination [17]. This, in turn, might affect adherence on ART, increasing their chances of virological (hence treatment) failure.

Patient viral load must necessarily be interpreted with caution - viral load is affected by inter-patient variation, laboratory errors, opportunistic infections, pre-ART viral load and the ART regimen [18, 19]. These factors can cause transient viral ‘blips’ which could be misinterpreted as virological failure. After such blips, viral load spontaneously drops to undetectable levels without change in ART regimen. It is possible that any of these factors may have influenced the findings reported in this paper.

### Study limitations and strengths

An important study limitation is that the dataset analysed was based on patients with suspected treatment failure only and hence the findings cannot be generalized to the general population of PLHIVs in care. A further limitation is that this study investigated over-diagnosis of treatment failure in the absence of viral load testing. In the absence of viral load monitoring, this study cannot investigate the full scale of true treatment failure. Finally, incomplete documentation of viral load data was a notable challenge. This could possibly be due to clinicians failing to update all the patient details at the time of requesting for the viral load test. This limitation can be overcome through mentorship sessions and on-job training.

A key strength of this study is the availability of a large sample size, making it possible to analyze variables at bivariate and multivariate levels. Data quality was high and obtained entirely from the online viral load monitoring system in Kenya.

The findings from this study add to the body of knowledge on clinical and laboratory challenges of managing HIV-infected patients in low-resource settings, and build the case for the need for viral load monitoring of patients on ART.

## Conclusion

Clinical and immunological criteria when applied without viral load testing are not sufficient to definitively identify treatment failure in a timely manner. There is need for accurate treatment failure diagnosis based on viral load to avoid early or delayed switching of patients to second-line ART, and the subsequent consequences of that untimely switching. This study recommends universal viral load testing for all HIV-infected individuals in line with Kenya’s ART guidelines. Findings from this study further strengthen policy and guidelines on viral load testing as the standard reliable test to be conducted prior to switching patients from first to second line ART regimens. A viral suppression survey with genotypic resistance is needed in the future to shed more light on treatment failure.

## Abbreviations

3TC: Lamivudine
ABC: Abacavir
AMPATH: Academic Model Providing Access to Healthcare
APHIA*PLUS*: AIDS Population and Health Integrated Assistance; People Led, people owned Leadership in all structures, Universal access to services and Sustainability.
ART: Antiretroviral therapy
ATV: Atazanavir
AZT: Zidovudine
DBS: Dried blood spots
EFV: Efavirenz
HTS: HIV testing services
IAS: International AIDS society
KEMRI: Kenya Medical Research Institute
LPY/r: Lopinavir/Rotonavir
MOH: Ministry of Health
NHRL: National HIV reference laboratory
NVP: Navirapine
OVC: Orphaned and vulnerable children
PLHIV: People Living with the human immunodeficiency virus
PMTCT: Prevention of mother-to-child transmission
RT-PCR: Reverse transcription polymerase chain reaction
TDF: Tenofovir
VL: Viral Load
WHO: World Health Organization
